# Influence of a Whisper Loading Test on the Vibration Mechanism of the Vocal Folds in the Context of Organic Dysphonia

**DOI:** 10.3390/jcm15051735

**Published:** 2026-02-25

**Authors:** Rebekka Hoppermann, Jonas Kirsch, Theresa Pilsl, Marie Köberlein, Michael Döllinger, Matthias Echternach

**Affiliations:** 1 Division of Phoniatrics and Pediatric Audiology, Department of Otorhinolaryngology, University Hospital, Ludwig-Maximilian-University (LMU) Munich, Marchioninistrasse 15, 81377 Munich, Germany; rebekka.hoppermann@med.uni-muenchen.de (R.H.); jonas.kirsch@med.uni-muenchen.de (J.K.); theresa.pilsl@med.uni-muenchen.de (T.P.); marie.koeberlein@med.uni-muenchen.de (M.K.); 2 Clinic for Anesthesiology and Intensive Care Medicine at Charité, University Medicine Berlin, Charitéplatz 1, 10117 Berlin, Germany; 3 Antonio Salieri Department of Vocal Studies and Vocal Research in Music Education, mdw University of Music and Performing Arts Vienna, Anton-von-Webern-Platz, 1030 Vienna, Austria; 4 Division of Phoniatrics and Pediatric Audiology, Department of Otorhinolaryngology Head & Neck Surgery, University Hospital Erlangen, Friedrich-Alexander-University Erlangen-Nürnberg, Waldstrasse 1, 91054 Erlangen, Germany; michael.doellinger@uk-erlangen.de

**Keywords:** organic dysphonia, whisper loading task, high-speed videoendoscopy, GAW, EGG, AVQI, DSI, RBH, VHI

## Abstract

**Background/Objectives:** Whispering has frequently been recommended to patients in order to avoid vocal overuse, particularly in those with organic dysphonia. However, there is controversy as to whether whispering may negatively affect vocal function by promoting malregulatory phonatory patterns. The aim of this study was to investigate whether a standardized short-term whisper loading task induces measurable changes in vocal function and vocal fold vibration characteristics in patients with organic dysphonia. **Methods:** Eight patients with clinically diagnosed vocal fold mass lesions scheduled for phonosurgery were examined before and immediately after a 10 min forced whisper loading task. Vocal function was assessed using the Dysphonia Severity Index. Vocal fold vibration and phonatory characteristics were analyzed using transnasal high-speed videolaryngoscopy, electroglottography, and acoustic measures. Pre–post differences were evaluated using non-parametric statistical testing with Bonferroni correction. **Results:** All participants completed the whisper loading task, although most were unable to consistently reach the target sound pressure level. No statistically significant pre–post differences were found in DSI, acoustic measures, perturbation parameters, or glottal vibration indices derived from high-speed recordings and electroglottography. Pathological vibration patterns related to the underlying mass lesions were present but did not show systematic changes following whisper loading. **Conclusions:** In patients with organic dysphonia, the short-term forced whisper loading task did not result in statistically significant alterations of vocal function or vocal fold vibration patterns. These findings suggest that short-term whispering does not acutely exacerbate biomechanical or vibratory impairments in organic vocal fold pathology.

## 1. Introduction

Voice production is a highly complex neuromuscular and biomechanical process that depends on the integrity of the vocal fold tissue, precise motor control, and stable aerodynamic conditions [1]. Disturbances at any level of the voice production system may result in dysphonia, which clinically manifests as hoarseness, a reduced range of fundamental frequency (*f*_o_), decreased sound pressure level (SPL), reduced intensity of the overtone spectrum and vocal endurance, as well as associated symptoms such as coughing, breathing problems and dysphagia [2,3]. Dysphonia significantly affects communication and quality of life and is particularly relevant in professional voice users and patients with structural or organic laryngeal pathologies [4,5,6,7,8].

Organic dysphonia, as characterized by vocal fold mass lesions and/or impairment of laryngeal nerve function, may have different causes. In addition to injury to the recurrent nerve as a main cause for nerve-function-related organic dysphonia, different kinds of mass lesions might influence both histopathological and structural alterations of the vocal folds. Concerning the latter, there is frequently a histopathological and/or clinically based differentiation of vocal fold nodules, polyps, cysts, scarring, Reinke’s edema, or inflammatory changes [9]. These conditions may directly alter the biomechanical properties of the vocal fold cover, transition and body, thereby affecting vibration patterns and glottal closure [10]. As a consequence, vocal fold oscillation has frequently been found to be irregular and asymmetric, and voice production inefficient, often accompanied by increased phonatory effort and reduced vocal quality in patients with organic dysphonia [11].

It has been assumed that mass lesions, on the one hand, can result from increased stress to the vocal folds but, on the other hand, change stress characteristics during oscillation when present [12]. Mechanical stress during phonation is primarily characterized by collision forces, shear stress, and cyclic strain within the lamina propria [13].

Because of this association with vocal stress, in patients suffering from organic dysphonia it has been frequently recommended to avoid vocal overuse, both during the organic dysphonia, but also immediately after phonosurgery. Therefore, many patients use whispering for communication. In contrast to voiced phonation, whispering is an aero-acoustic mode of sound production characterized by the absence of regular vocal fold oscillation, incomplete glottal closure, and broadband noise generation through turbulent airflow [14]. Subglottal pressure during whispering differs typically from subglottal pressure during modal phonation, depending on the type of whisper [15]; however, specific laryngeal configurations, particularly during forced or tensioned whispering, may involve increased intrinsic laryngeal muscle activation and supraglottic constriction [16,17].

Clinically, the use of whispering remains a matter of debate, particularly in voice therapy and postoperative voice management [18]. Although whispering is commonly regarded as a protective “voice-saving” strategy, several studies indicate that specific forms of whispering may promote hyperfunctional/malregulative laryngeal behavior, increased ventricular fold involvement, and potentially heightened mechanical stress on the vocal folds [16,17]. As a result and pointed out before, whispering is frequently discouraged after phonosurgery, although empirical evidence supporting this recommendation remains limited and partly contradictory [19].

Recent experimental work has attempted to quantify the effects of whispering using standardized whisper loading protocols analogous to traditional vocal loading tests [20]. For vocally healthy subjects, it has been demonstrated that a short-term forced whisper loading task resulted in only minor changes in vocal function and vocal fold vibration, as assessed by high-speed videolaryngoscopy, electroglottography, and acoustic measures [20]. Notably, slight increases in minimum intensity, Glottal Gap Index, and glottal open quotient were observed, particularly in subjects producing a tensioned whisper, suggesting subtle alterations in laryngeal configuration and phonatory efficiency [20].

However, these findings are derived exclusively from vocally healthy individuals. The transferability of these results to patients with organic dysphonia therefore remains unclear. It could be expected that structural vocal fold alterations may fundamentally change how whispering-related aerodynamic forces are distributed across the tissue, potentially increasing vulnerability to shear stress, edema, or maladaptive vibratory patterns [10,21]. In vocal folds affected by scarring or inflammation, even phonatory behaviors without regular oscillation may elicit unfavorable biomechanical reactions that do not occur in healthy tissue [22,23,24].

Given the ongoing clinical debate regarding the safety of whispering and the lack of data focusing on pathological voices, a systematic prospective investigation of the influence of a whisper stress test on vocal fold vibration mechanisms in organic dysphonia is warranted. High-speed videolaryngoscopy provides a unique opportunity to analyze fine-grained vibratory behavior, including glottal area dynamics, phase symmetry, and closure patterns, which are particularly sensitive to tissue alterations [25].

Therefore, the aim of the present study was to examine whether a standardized whisper loading test induces measurable changes in vocal fold vibration characteristics and vocal function in patients suffering from organic dysphonia. It was hypothesized that, compared to healthy voices, individuals with organic vocal fold pathology will exhibit more pronounced alterations in vibratory parameters following whisper loading, reflecting reduced biomechanical resilience of the vocal fold tissue.

## 2. Materials and Methods

After approval from the local ethics committee (Medical Ethics Committee of the University of Munich, No. 20/282), 8 subjects with organic dysphonia (age range 28–65 years; 7 females, 1 male) were included (Table 1). All participants were, after clinical diagnosis, scheduled for phonosurgery and provided written informed consent prior to inclusion to the experiment. Individuals with known pregnancy were excluded from participation.

A. Task

The participants were asked first to produce all phonations required for the establishment of the Dysphonia Severity Index (DSI), i.e., softest phonation, highest *f*_o_ and maximum phonation time (MPT) on comfortable pitch and loudness, which was also used for the calculation of the jitter [29]. All vocalizations were performed on the/a/vowel. Acoustic Voice Quality Index (AVQI) measurements were acquired via VOXplot (version 2.6.0; Jörg Mayer). Subsequently, the subjects were asked to perform a whisper loading test, according to previous studies [20]. Here, the participants were asked to whisper the German text “Das tapfere Schneiderlein” (Grimm brothers) at 70 dB(A), measured by a SPL meter (lingWAVES SPL meter II, Wevosys, Bamberg, Germany) in 30 cm distance to the mouth. The loading test was recorded using lingWAVES software (version 3.2; Wevosys, Bamberg, Germany). An arrow appeared on the screen when the 70 dB(A) criterion was not met, indicating that the subject should increase the SPL. Prior to the loading test and immediately after, the subjects were asked to sustain a phonation on the vowel/i/at a determined pitch (*f*_o_ ≈ 125 Hz (B2) for male and *f*_o_ ≈ 250 Hz (B3) for female participants) for the high-speed videoendosycopy (HSV) recording, see below. Also, after vocal loading, there was a repetition of the DSI measurement. Table 2 provides an overview of the exact sequence of tasks.

B. Recordings

The participants’ sustained phonations on the vowel/i/were recorded simultaneously using transnasal HSV, electroglottography (EGG) and an external microphone, in accordance with protocols described in prior studies [20]. The vowel/i/was used due to the best laryngoscopic view to the vocal folds. HSV yields a two-dimensional visualization of vocal fold vibration, whereas EGG provides information on the three-dimensional vocal fold contact pattern by measuring variations in electrical impedance.

Sound pressure level calibration of the microphone (DPA 4061, DPA Microphones, Kokkedal, Denmark), measured over time during HSV, was performed using the Sopran software (version 1.0.29; Svante Granqvist, Karolinska, Sweden) in combination with an SPL meter (Tecpel, Taipei, Taiwan). EGG signals were captured with an EG2-PCX2 device (Glottal Enterprises, Syracuse, NY, USA). HSV recordings were obtained transnasally using a Fastcam SA-X2 system (Photron, Tokyo, Japan) in combination with a flexible endoscope (ENF-GP, Olympus, Hamburg, Germany). Recordings were performed at a frame rate of 20,000 frames per second with a monochrome image resolution of 386 × 320 pixels. HSV recordings were subsequently post-processed by rotation, Fourier-based filtering, and cropping in accordance with previously established procedures [30].

C. Measures

The HSV data were segmented using the Glottal Analysis Tools (GAT) software (version 2025; University Hospital at FAU Erlangen–Nürnberg, Germany) [31], estimating the time-varying lateral deflection of the vocal fold edges along the anterior–posterior axis of the glottis [32]. Based on the extracted glottal area waveform, the open quotient (OQ_GAW_), the Glottal Gap Index (GGI) [20] and the closing quotient (ClQ_GAW_) were calculated for the specified analysis windows using Vocaliscope (version 3.8; Jonas Kirsch, LMU University Hospital Munich, Germany). For glottal quotient computations, a tolerance threshold of 5% was applied, such that Glottal Area Waveform (GAW) values exceeding 5% of the baseline (corresponding to the pixel count of the maximally open glottis) were classified as open, whereas values ≤ 5% were interpreted as an indication of a closed glottis. The OQ_EGG_ was calculated according to the algorithms introduced by Howard [33].

More information on calculated parameters can be found in Table 3.

D. Statistical Evaluation

Parameters obtained from each participant at two time points were subjected to statistical analysis using MATLAB (version 25.1.0, R2025a; The MathWorks Inc., Natick, MA, USA). Differences in open and closing quotients were evaluated using the two-tailed, non-parametric Wilcoxon signed-rank test (exact method). The significance level was set at α = 0.05. *p*-values were adjusted for multiple comparisons using the Bonferroni correction (m = 12), due to 12 different parameters from each subject. In addition, the rank-serial effect size was calculated and its 95% confidence interval using bootstrapping [34,35] with *N* = 10,000 artificial data sets and bias corrected and accelerated percentile method [36,37].

## 3. Results

**Figure 1:**
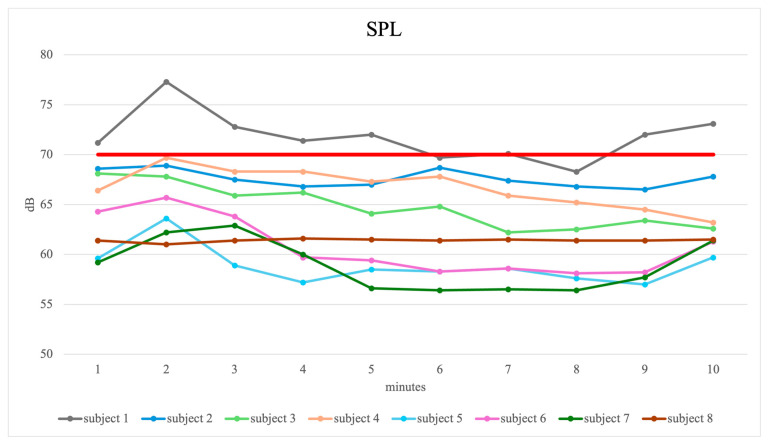
Mean SPL for each minute of the whisper loading test for the 8 subjects. The 70 dB(A) criterion line is marked in bold red.

All subjects completed the whisper loading test without interruption. However, as shown in Figure 1 and Table 4, all subjects except subject 1 did not reach the target SPL of 70 dB(A). Subject 1 matched in 80% of the 10 min intervention of this criterion with a median SPL of 71.7 dB(A). All remaining subjects showed considerably lower values with a median mean SPL of 63.1 dB(A).

When comparing the mean values over time of *f*_o_, SPL, GGI, Cepstral Peak Prominence (CPP), Harmonic-to-Noise Ratio (HNR), Relative Average Perturbation (RAP), ClQ, OQ*_Source_*, DSI (and its calculation descendants) between pre and post recordings using the Wilcoxon test and Bonferroni correction, no significant *p*-values arose at all. Furthermore, all *p*-values and *p*-values after Bonferroni correction had a far distance from the level of statistical significance (Table 5 and Table 6). Since all effect sizes are highly variable, they were not discussed further.

More in detail, the DSI was—in general—measured at a rather low value. The single components showed a high SPL for the lowest intensity, a low highest *f*_o_ and a short MPT. However, these values did not change statistically significantly after the whisper loading test.

**Figure 2:**
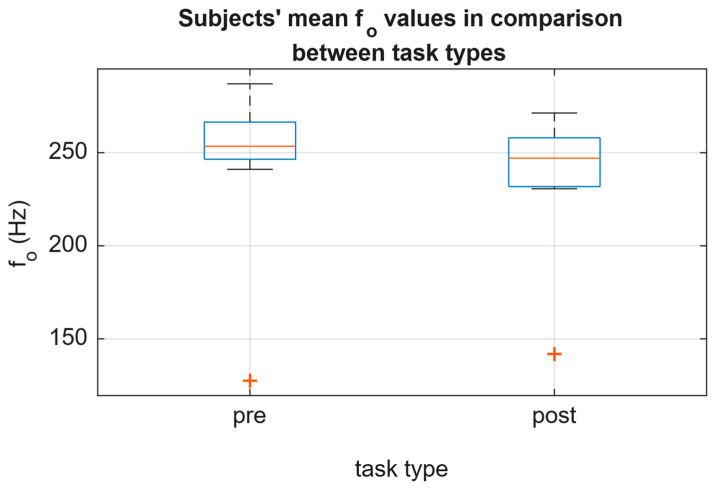
Subjects’ mean *f*_o_ values sorted by task type. Median *f*_o_ values decrease to a negligible degree (9 Hz).

**Figure 3:**
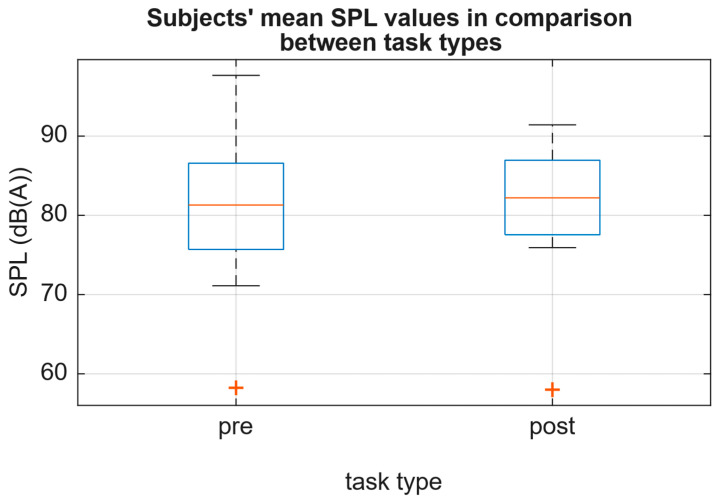
Subjects’ mean SPL values sorted by task type. The medians do not change significantly between pre and post tasks.

The median of the group’s mean *f*_o_, as well as median of the group’s mean SPL, did not change significantly between pre and post recordings (change by −5 Hz and +0.9 dB(A); Figure 2 and Figure 3).

**Figure 4:**
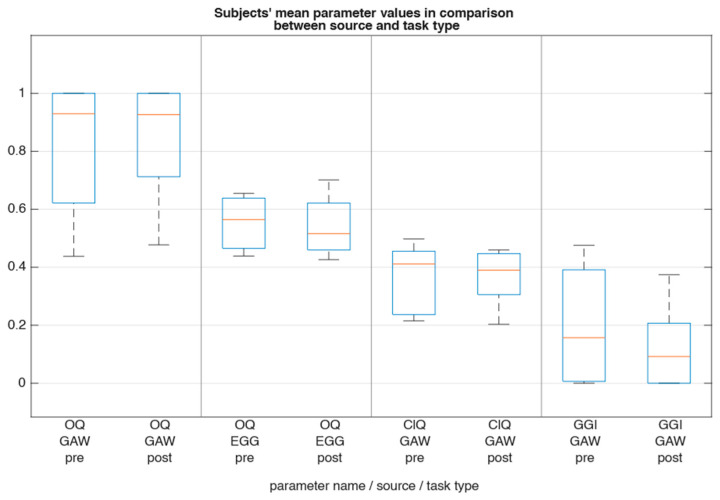
Subjects’ mean OQ_GAW_, OQ_EGG_, ClQ and GGI values sorted by task and source type. OQ_EGG_, ClQ and GGI decrease slightly in the post task.

**Figure 5:**
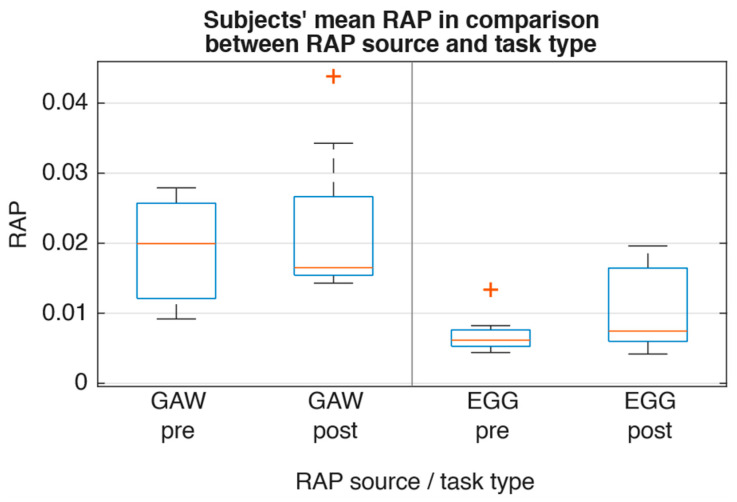
Subjects’ mean RAP values sorted by task and source type.

**Figure 6:**
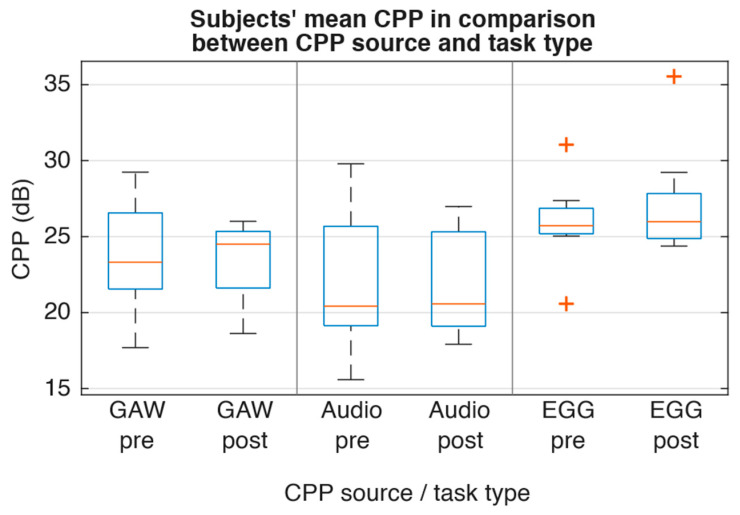
Subjects’ mean CPP values sorted by task and source type.

**Figure 7:**
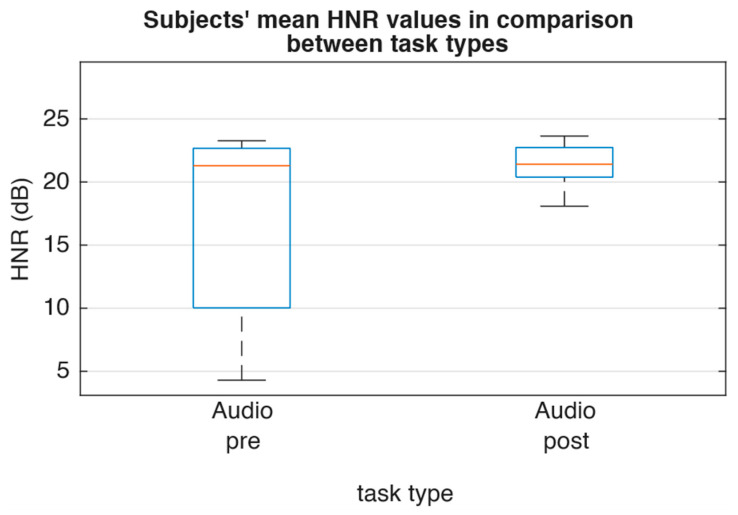
Subjects’ mean HNR values sorted by task type.

For the glottal parameters, Figure 4 showed that there was only a slight decrease in median OQ_EGG_ (−0.04), ClQ_GAW_ (−0.02), and GGI (−0.07). Frequency perturbation as described by RAP exhibited minor magnitude and contrary direction (RAP_GAW_ −0.004; RAP_EGG_ +0.001; Figure 5). Furthermore, spectral comparison of audio recordings by means of CPP and HNR (Figure 6 and Figure 7) showed no changes between pre and post recordings.

**Figure 8:**
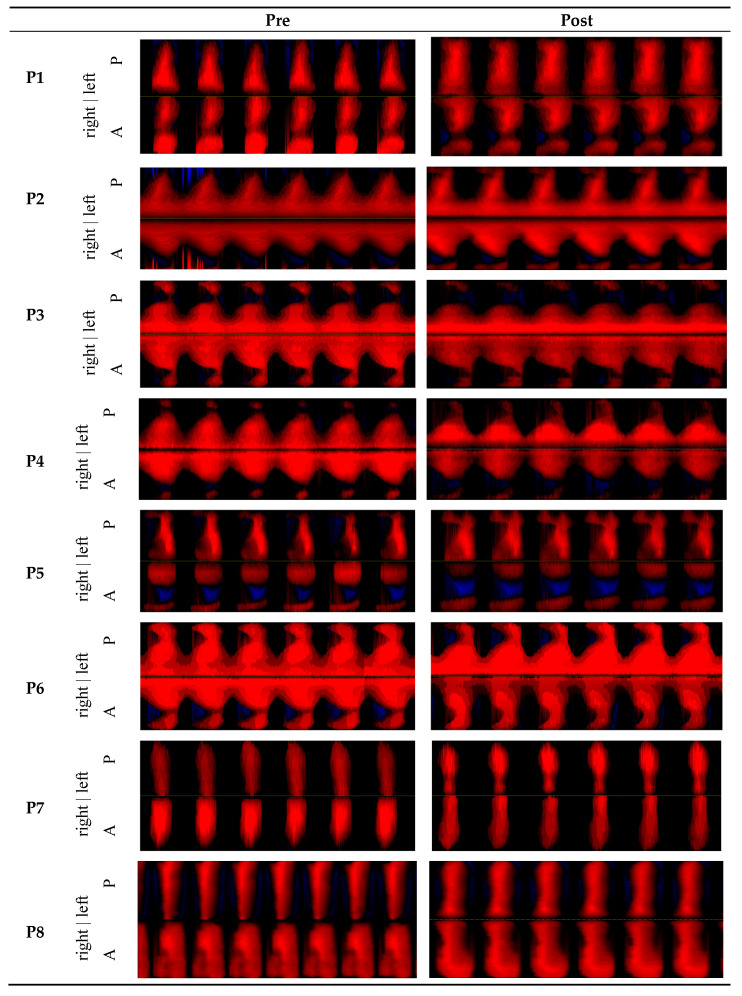
Phonovibrograms (PVG) representing pre and post recordings for all subjects. The intensity of the red color corresponds to the distance from the glottis midline, the blue refers to a crossing over the glottis midline to the other side. A = anterior, P = posterior.

The Phonovibrograms (PVGs) exhibited strong abnormalities due to the mass lesions (Figure 8). Here, for some subjects there was no oscillation at the mass lesion, i.e., subjects P1, P3, P5 and P6. Further, for some subjects there were some anterior–posterior or left–right phase differences. However, there were no great detectable changes concerning periodicity or changes in the pre-post comparison.

## 4. Discussion

The presented study analyzed the influence of forced whispering on vocal fold oscillation patterns and vocal outcome, expressed by the DSI, in patients with vocal fold mass lesions. In general, it has been found that such whisper loading neither affected the vocal fold oscillation pattern nor the vocal outcome.

It has frequently been hypothesized that whispering may impair vocal function [16,17]. This concern does not only apply to vocally healthy individuals but is considered particularly relevant for patients suffering from organic dysphonia. Moreover, the potential impact of whispering following phonosurgical interventions has been repeatedly discussed. However, previous studies in vocally healthy subjects have demonstrated that a defined, short-term whispering task affects vocal function only to a small extent [20].

In the presented study, a cohort of patients with organic dysphonia was analyzed. Baseline measurements of the DSI, as well as other characteristic parameters, revealed pronounced impairments of vocal performance in comparison to healthy subjects [38]. Specifically, baseline DSI values were markedly reduced, while the AVQI was substantially increased [39]. Examination of the DSI components demonstrated notable alterations: MPT was considerably shorter than that observed in vocally healthy individuals, and the maximum *f*_o_ was reduced. Most prominently, minimum intensity was significantly increased. As minimum intensity is associated with phonation threshold pressure (PTP) [40], it can be assumed that mass-related alterations of the vocal folds in organic dysphonia may result in inefficient transfer of subglottal pressure into vibratory energy.

Because of the mass lesions and the associated mass difference between the vocal folds, it could be speculated that the irregularities might be increased on the baseline. However, although the PVG showed many pathological oscillation patterns, the analyzed data failed to exhibit strong impairments on periodicity. In accordance with this, the jitter during the DSI evaluation, the RAP and CPP did not show a general increase compared to healthy subjects [20]. Interestingly, these data concerning periodicity and perturbation contradict the perceptive measurements of the subjective Roughness–Breathiness–Hoarseness (RBH) scale prior to the experiments, in which the roughness was increased for all subjects. It has been shown that the roughness is frequently associated with a rise in aperiodicities and/or perturbation measures [41]. Thus, it might be that the patients compensated vocal function by a greater amount when focusing on the experiment.

The fact that neither the overall DSI nor additional acoustic parameters such as HNR or CPP showed significant changes following the whispering task may be attributed to several factors. First, many patients already exhibited a certain degree of vocal breathiness at baseline, as reflected in the RBH scale and the reduced Glottal-to-Noise-Excitation-Ratio (GNE) values, recall Table 1. Consequently, these voices may already tend toward a whisper-like phonatory pattern even when attempting normal phonation and might be used to a phonation in direction to whisper for a long time. Second, the target vocal intensity of 70 dB(A) could be maintained for a substantially shorter proportion of subjects compared to vocally healthy subjects in the previous study [20]. It thus remains unclear whether the lack of significant changes is attributable to the whispering intervention itself or rather to the characteristics of the investigated cohort. Also in this context, it should be mentioned that the subjects were asked to produce a tensioned version of the whispering assuming that a strong effect on vocal fold oscillation pattern would be more likely in this version. However, it cannot be totally excluded that an untensioned whispering would exhibit different data.

The OQ_GAW_ in all subjects was very high and disagreed with OQ_EGG_. As has been demonstrated in many studies [42], this was not unexpected. In OQ_GAW_ greater than 0.7 there is frequently a great disagreement for these both values because of the fact that the EGG still shows impedance changes when the glottis exhibits a gap throughout the glottal cycle.

A frequently and controversially discussed issue concerns the question of whether whispering is harmful to vocal function. It must be emphasized that the present data merely indicate that a short-term whispering intervention does not lead to significant changes in vocal function. The study does not address whether prolonged or habitual whispering might induce functional alterations that could further reduce vocal performance in addition to the underlying organic dysphonia. Furthermore, it remains to be questioned whether whispering could influence the organic pathology itself, thus changing size or morphology. However, it should be noted that whispering is characterized by the absence of vocal fold oscillation, and therefore the impact/shear stress on the tissue is expected to be minimal. Also in this respect, a limitation of this study is the fact that whispering was not documented endoscopically. This was deliberately omitted to avoid artificially altering the whispering task. Nevertheless, it remains unclear whether whispering in patients with organic dysphonia corresponds to the typical posterior glottal gap observed in vocally healthy individuals [14] or whether airflow turbulence predominantly arises at the site of the organic lesion itself.

There are further limitations of this study. First is that the vowel condition differed for the calculation of the DSI and laryngoscopy. The vowel/i/was used for laryngoscopy due to the best visibility to the vocal folds. However, to avoid vocal tract/vocal fold interactions in vowels with low resonance frequency, usually for multiple voice diagnostics the/a/vowel has been used [43]. Thus, this vowel difference has been accepted in the study design. Still, it cannot be excluded that the experiment would show different data when executed on different vowel qualities.

Another limitation of the present study is the sex imbalance within the cohort, with a higher proportion of female patients (*n* = 7) than male patients (*n* = 1). This overrepresentation reflects the known epidemiological pattern of voice disorders, where females are affected more frequently than males [44]. Several investigations have demonstrated that voice disorders, including organic dysphonia, occur significantly more often in women than in men, potentially due to anatomical, physiological, and sociocultural factors influencing vocal load and susceptibility to vocal pathology [44].

Regarding the analyzed cohort, it must be noted that various organic pathologies were grouped together. Certainly, morphology, shape and stiffness could contribute in a different way to the data. Future studies should aim to include larger sample sizes as well as a greater number of specific diagnostic subgroups. However, based on the present data, it appears uncertain whether an increased sample size alone would have resulted in statistically significant changes, as the observed *p*-values were far from statistical significance thresholds.

Finally, no secondary follow-up analysis was conducted (e.g., 10 min after the whisper loading task), limiting insight into potential short-term recovery effects. Because of the lack of statistically significant changes in the presented experiment, on the one hand, and due to the finding that effects on oscillation after short-term intervention appear frequently immediately after the intervention [45], on the other, it appears unlikely that strong changes could be expected in a follow-up examination.

## 5. Conclusions

In this study, no significant pre–post changes were observed in global voice measures or detailed vibratory parameters following a 10 min period of forced whispering in patients suffering from organic dysphonia. Despite clearly pathological vibration patterns, whisper loading did not further alter vocal fold oscillation characteristics or vocal performance. These findings indicate that a 10 min whisper task with an achieved median SPL of ~63 dB(A) (with only one subject reliably reaching 70 dB(A)) did not result in measurable acute changes concerning biomechanical or vibratory dysfunction in this patient group.

## Figures and Tables

**Table 1:** Data from the test subjects: age in years; sex (♀ = female, ♂ = male); medical diagnosis; affected vocal fold side (lateralization; r = right, l = left); VHI = Voice Handicap Index [26]; RBH = Roughness–Breathiness–Hoarseness Index [27]; AVQI = Acoustic Voice Quality Index [28]; GNE = Glottal-to-Noise-Excitation-Ratio; VC = Vital Capacity; Frequency Range; Dynamic Range; Presurgical laryngoscopic image.

Subject	1	2	3	4	5	6	7	8
Age (y)	56	43	31	45	53	28	65	61
Sex	♀	♀	♀	♀	♂	♀	♀	♀
Diagnosis	Polyp	Polyp, edema	Vocal fold thickening	Polyp, vocal fold thickening	Polyp	Vocal fold thickening	Vocal fold thickening	Polyp
Lateralization	r	r	l	r, l	r	r > l	l	r
VHI	49	29	62	67	58	55	62	85
RBH	1–0–1	2–0–2	2–2–2	2–2–2	1–0–1	1–1–1	1–0–1	2–2–2
AVQI	5.04	3.95	6.27	3.33	4.27	4.81	4.27	7.18
GNE	0.43	0.6	0.29	0.54	0.54	0.44	0.55	0.14
VC (L)	3.72	3.38	3.13	4.42	5.34	3.42	2.7	2.16
Frequency range (Hz)	542	431	348	392	205	209	283	189
Dynamic range (dB(A))	43	29	20	33	42	29	24	17
Presurgical laryngoscopic image	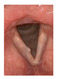	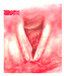	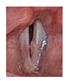	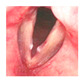	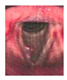	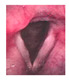	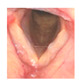	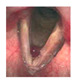

**Table 2:** Overview of the task sequence.

Order	Task	Fundamental Frequency
1.1	Dysphonia Severity Index (pre)	
1.2	High-speed examination (pre): sustained note at comfortable loudness (vowel/i/)	*f*_o_ ≈ 125 Hz (♂) *f*_o_ ≈ 250 Hz (♀)
2.1	Whisper loading task (>70 dB(A)) for 10 min, distance to SPL meter 30 cm	
3.1	Dysphonia Severity Index (post)	
3.2	High-speed examination (post): sustained note at comfortable loudness (vowel/i/)	*f*_o_ ≈ 125 Hz (♂) *f*_o_ ≈ 250 Hz (♀)

**Table 3:** Calculated parameters with abbreviation, source and additional information.

Parameter	Additional Information	Abbreviation	Source		
			GAW	EGG	Audio
Closing Quotient		ClQ*_Source_*	×		
Open Quotient	Howard method	OQ*_Source_*	×	×	
Relative Average Perturbation		RAP*_Source_*	×	×	
Sound Pressure Level	dB(A), 125 ms window	SPL			×
Fundamental Frequency	Pitch Estimation Filter method	*f*_o_	×		
Glottal Gap Index		GGI	×		
Harmonic-to-Noise Ratio	Based on cross-correlation	HNR			×
Cepstral Peak Prominence	Linear baseline fitting	CPP	×	×	×

**Table 4:** Median SPL in dB(A) and inter quartile range (IQR) per subject, describing the dose of the vocal loading test.

Dose/Subject	1	2	3	4	5	6	7	8
Median SPL (dB(A))	71.70	67.45	64.45	66.85	58.55	59.55	58.45	61.40
IQR	2.22	1.55	3.33	2.80	1.65	4.80	4.53	0.10

**Table 5:** Pre-post comparison with median values and IQR for the Dysphonia Severity Index (DSI), minimum intensity (I_min_), maximum phonation time (MPT), jitter and maximum fundamental frequency (*f*_max_). The *p*-values and the effect sizes refer to the Wilcoxon signed-rank test.

	Pre		Post		*p*-Value (Non-Corrected)	Effect Size (±2 STD)
	Median	IQR	Median	IQR		
DSI	1.55	2.45	1.3	0.95	0.31	0.44 (0.48)
I_min_ (dB(A))	54.05	4.2	55.3	5.93	0.46	0.33 (0.48)
MPT (s)	9.21	5.72	9.16	8.33	0.58	0.29 (0.38)
Jitter (%)	0.20	1.19	0.25	0.19	0.64	0.19 (0.36)
*f*_max_ (Hz)	437.12	143.6	418.15	231.82	0.38	0.39 (0.53)

**Table 6:** Pre-post comparison with median values for high-speed videoendoscopy (HSV) voice parameters. The *p*-values and the effect sizes refer to the Wilcoxon signed-rank test.

Group Median	Pre	Post	*p*-Value	*p*-Value (Corrected)	Effect Size (±2 STD)
*f*_o_ (Hz) (female only)	254.0120	248.8600	0.22	1	0.57 (0.54)
SPL (dB(A))	81.3026	82.2094	0.95	1	0.06 (0.34)
ClQ_GAW_	0.4111	0.3901	0.95	1	0.06 (0.33)
OQ_GAW_	0.9298	0.9266	0.63	1	0.33 (0.49)
OQ_EGG_	0.5644	0.5158	0.74	1	0.17 (0.35)
GGI	0.1569	0.0921	0.30	1	0.50 (0.53)
HNR (dB)	21.2935	21.4093	0.31	1	0.44 (0.47)
RAP_GAW_	0.0199	0.0165	0.31	1	0.44 (0.47)
RAP_EGG_	0.0062	0.0075	0.20	1	0.56 (0.55)
CPP_GAW_ (dB)	23.3163	24.5029	0.74	1	0.17 (0.35)
CPP_AUDIO_ (dB)	20.4229	20.5731	0.64	1	0.22 (0.60)
CPP_EGG_ (dB)	25.7189	25.9826	0.74	1	0.17 (0.35)

## Data Availability

The data presented in this study are available on request from the corresponding author.
